# Supplementary material for: Identification of photoperiod-induced specific miRNAs in the adrenal glands of Sunite sheep (Ovis aries)
Source: Front Vet Sci. 2022 Jul 22;9:888207. doi: 10.3389/fvets.2022.888207 (PMC9354845; doi:10.3389/fvets.2022.888207)
Supplement: Supplementary file 1 [file Data_Sheet_1.ZIP › Supplementary file/miRDeep2 file.zip!/miRDeep2 file/Know_Structure/LP42A3/oar-mir-30a.pdf]

miRBase precursor : oar-mir-30a
Total read count : 103785
oar-miR-30a-5p read count : 97466
oar-miR-30a-3p read count : 6319
remaining reads : 0

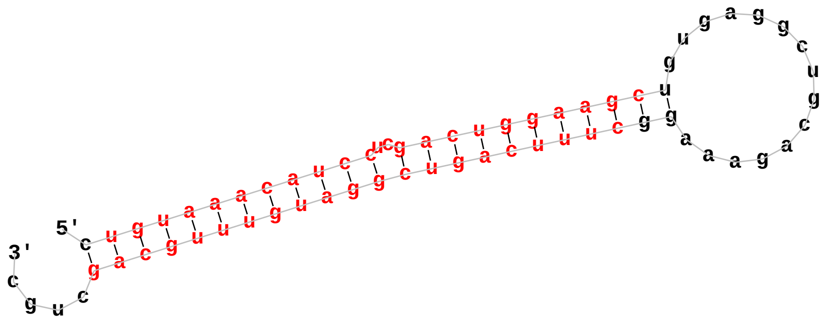

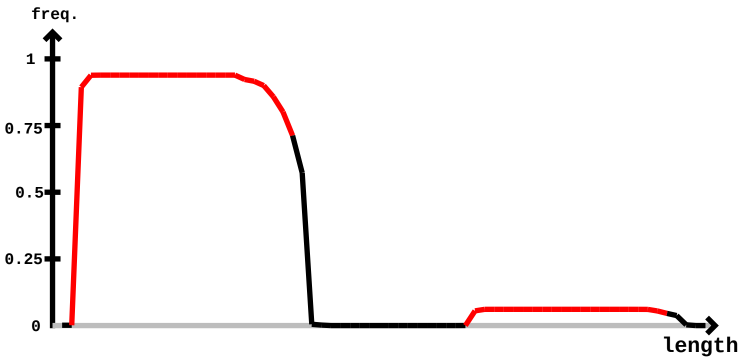


```
                                                oar-miR-30a-3p
     oar-miR-30a-5p
5'- cuguaaacauccucgacuggaagcugugaggcugcagaaaggcuuucagucggauguuugcagcugc   -3'    exp
    ((((((((((((..(((((((((((...............)))))))))))))))))))))))....    reads      mm         sample
    cuguaaacauccuUgacu.................................................    2          1          seq
    cuguaaacauccucgacugg...............................................    5          0          seq
    cuguaaacauccuUgacugg...............................................    1          1          seq
    cuguaaacauccucgacugga..............................................    15         0          seq
    cuguaaacauccuUgacugga..............................................    1          1          seq
    cuguaaacauccucgacuggaa.............................................    12         0          seq
    cuguaaacauccucgacuggaag............................................    8          0          seq
    cuguaaacauccuUgacuggaag............................................    3          1          seq
    cuguaaacauccucgacuggaagc...........................................    30         0          seq
    cuguaaacauccucgacuggaagcu..........................................    26         0          seq
    cuguaaacauccuUgacuggaagcu..........................................    21         1          seq
    .uguaaacauccuUgacu.................................................    1488       1          seq
    .uguaaacauccucgacu.................................................    202        0          seq
    .uguaaacauccucUacu.................................................    1          1          seq
    .uguaaacauccucgacug................................................    166        0          seq
    .uguaaacauccuUgacug................................................    541        1          seq
    .uguaaacauccuUgacugg...............................................    864        1          seq
    .uguaaacauccucgacugg...............................................    794        0          seq
    .uguaaacauccuUgacugga..............................................    775        1          seq
    .uguaaacauccucgacugga..............................................    3557       0          seq
    .uguaaacauccuUgacuggaa.............................................    1014       1          seq
    .uguaaacauccucgacuggaa.............................................    4853       0          seq
    .uguaaacauccuUgacuggaag............................................    3156       1          seq
    .uguaaacauccucgacuggaag............................................    6017       0          seq
    .uguaaacauccucgacuggaagc...........................................    11992      0          seq
    .uguaaacauccuUgacuggaagc...........................................    2394       1          seq
    .uguaaacauccuUgacuggaagcu..........................................    31461      1          seq
    .uguaaacauccucgacuggaagcu..........................................    23277      0          seq
    .uguaaacauccuUgacuggaagcug.........................................    52         1          seq
    .uguaaacauccucgacuggaagcug.........................................    22         0          seq
    .uguaaacauccuUgacuggaagcugu........................................    30         1          seq
    .uguaaacauccucgacuggaagcugu........................................    2          0          seq
    ..guaaacauccuUgacug................................................    1          1          seq
    ..guaaacauccuUgacugg...............................................    2          1          seq
```

```
                                          oar-miR-30a-3p
 oar-miR-30a-5p
cuguaaacauccucgacuggaagcugugaggcugcagaaaggcuuucagucggauguuugcagcugc

..guaaacauccuUgacugga..............................................    8      1      seq
..guaaacauccucgacugga..............................................    3      0      seq
..guaaacauccucgacuggaa.............................................    8      0      seq
..guaaacauccuUgacuggaa.............................................    5      1      seq
..guaaacauccuUgacuggaag............................................    25     1      seq
..guaaacauccucgacuggaag............................................    8      0      seq
..guaaacauccuUgacuggaagc...........................................    39     1      seq
..guaaacauccucgacuggaagc...........................................    27     0      seq
..guaaacauccucgacuggaagcu..........................................    229    0      seq
..guaaacauccuUgacuggaagcu..........................................    3903   1      seq
..guaaacauccuUgacuggaagcug.........................................    219    1      seq
..guaaacauccuUgacuggaagcugu........................................    157    1      seq
...uaaacauccucgacuggaagcu..........................................    12     0      seq
...uaaacauccuUgacuggaagcu..........................................    12     1      seq
...uaaacauccuUgacuggaagcug.........................................    16     1      seq
...uaaacauccuUgacuggaagcugu........................................    9      1      seq
.....aacauccuUgacuggaagcu..........................................    1      1      seq
........................................Agcuuucagucggauguuu........    1      1      seq
.........................................gcuuucagucggauguuu........    1      0      seq
.........................................gcuuucagucggauguuuAc......    4      1      seq
.........................................gcuuucagucggauguuuAca.....    7      1      seq
.........................................gcuuucagucggauguuuAcag....    2      1      seq
.........................................gcuuucagucggauguuuAcagc...    8      1      seq
..........................................cuuucagucggauguuu........    7      0      seq
..........................................cuuucagucggauguuuA.......    15     1      seq
..........................................cuuucagucggauguuug.......    4      0      seq
..........................................cuuucagucggauguuuAc......    479    1      seq
..........................................cuuucagucggauguuugc......    103    0      seq
..........................................cuuucagucggauguuuAca.....    818    1      seq
..........................................cuuucagucggauguuugca.....    87     0      seq
..........................................cuuucagucggauguuuAcag....    603    1      seq
..........................................cuuucagucggauguuugcag....    193    0      seq
..........................................cuuucagucggauguuuAcagc...    2299   1      seq
..........................................cuuucagucggauguuugcagc...    1007   0      seq
..........................................cuuucagucggauguuugcagcu..    76     0      seq
...........................................uuucagucggauguuuAc......    27     1      seq
...........................................uuucagucggauguuugc......    5      0      seq
...........................................uuucagucggauguuuAca.....    21     1      seq
...........................................uuucagucggauguuugca.....    7      0      seq
...........................................uuucagucggauguuuAcag....    15     1      seq
...........................................uuucagucggauguuugcag....    22     0      seq
...........................................uuucagucggauguuuAcagc...    153    1      seq
...........................................uuucagucggauguuugcagc...    136    0      seq
...........................................uuucagucggauguuugcagcu..    212    0      seq
...........................................uuucagucggauguuugcagcug.    3      0      seq
............................................uucagucggauguuugcag....    1      0      seq
............................................uucagucggauguuugcagcu..    2      0      seq
.............................................ucagucggauguuugcagc...    1      0      seq
```
